# Catechol-*O*-methyltransferase val^158^met Polymorphism Interacts with Sex to Affect Face Recognition Ability

**DOI:** 10.3389/fpsyg.2016.00965

**Published:** 2016-06-27

**Authors:** Yvette N. Lamb, Nicole S. McKay, Shrimal S. Singh, Karen E. Waldie, Ian J. Kirk

**Affiliations:** School of Psychology, The University of AucklandAuckland, New Zealand

**Keywords:** neurogenetics, COMT, recognition, gene-sex interaction, memory

## Abstract

The catechol-*O*-methyltransferase (*COMT*) val158met polymorphism affects the breakdown of synaptic dopamine. Consequently, this polymorphism has been associated with a variety of neurophysiological and behavioral outcomes. Some of the effects have been found to be sex-specific and it appears estrogen may act to down-regulate the activity of the COMT enzyme. The dopaminergic system has been implicated in face recognition, a form of cognition for which a female advantage has typically been reported. This study aimed to investigate potential joint effects of sex and *COMT* genotype on face recognition. A sample of 142 university students was genotyped and assessed using the Faces I subtest of the Wechsler Memory Scale – Third Edition (WMS-III). A significant two-way interaction between sex and *COMT* genotype on face recognition performance was found. Of the male participants, *COMT* val homozygotes and heterozygotes had significantly lower scores than met homozygotes. Scores did not differ between genotypes for female participants. While male val homozygotes had significantly lower scores than female val homozygotes, no sex differences were observed in the heterozygotes and met homozygotes. This study contributes to the accumulating literature documenting sex-specific effects of the *COMT* polymorphism by demonstrating a *COMT*-sex interaction for face recognition, and is consistent with a role for dopamine in face recognition.

## Introduction

Faces are a ubiquitous feature of our social environment. Generally, research has documented a female advantage for remembering previously encountered faces (Herlitz and Lovén, 2013). The dopaminergic system has been implicated in face recognition (e.g., Rypma et al., 2015) and dopamine availability in the mammalian central nervous system is affected by the catechol-*O*-methyltransferase (COMT) enzyme (Egan et al., 2001). Activity of the COMT enzyme is affected by variation in the gene coding for the COMT, which includes the *COMT* val^158^met polymorphism (Chen et al., 2004). COMT activity is also regulated by oestrogen (e.g., Jiang et al., 2003), and certain effects of the *COMT* polymorphism on cognition appear to be sex-specific (e.g., Barnett et al., 2007; Soeiro-De-Souza et al., 2013; Gurvich and Rossell, 2015). This study investigated the potential sex-specific effects of the *COMT* val^158^met polymorphism on memory for faces in young adults.

Sex differences are commonly reported for a range of cognitive functions and their underlying neural substrates. Numerous studies involving face recognition tasks have reported that women recognize significantly more faces than men (e.g., O’Toole et al., 1998; Guillem and Mograss, 2005; Rehnman and Herlitz, 2007). Although sex differences have not been invariably found (e.g., Haut and Barch, 2006; van Wingen et al., 2010), a recent meta-analysis by Herlitz and Lovén (2013) noted a significant overall effect of sex on memory for faces. This female advantage was present at similar magnitudes in children, adolescents and adults (Herlitz and Lovén, 2013). At the neuroanatomical level, girls and women show larger fusiform face areas (FFAs) than boys and men (e.g., Golarai et al., 2009). The FFA is a face-specific region of the fusiform gyrus, an integral component of the face processing network (Sergent and Signoret, 1992; Kanwisher et al., 1997; Gobbini and Haxby, 2007). Larger FFAs have in turn been linked to higher levels of face recognition performance (e.g., Golarai et al., 2009; Furl et al., 2011).

The biopsychosocial model of sex differences in cognition describes biological and environmental factors as being intertwined, interacting with each other to affect cognitive phenotypes (Halpern, 2012). Early perceptual experiences have been suggested to contribute to females developing a higher level of expertise in recognizing faces (Herlitz and Lovén, 2013). Sex hormones may also play a role in memory-related sex differences, with estrogen affecting brain regions involved in learning and memory (Pompili et al., 2012) and enhancing the release of striatal dopamine (Becker, 1990; Becker and Rudick, 1999). In young women, a single dose of progesterone decreases activity in the fusiform gyrus and amygdala during the encoding of faces, resulting in poorer subsequent face recognition (van Wingen et al., 2007). Estradiol is positively associated with face recognition performance in females while no association is seen in males (Yonker et al., 2003).

The dopaminergic system appears to influence fusiform gyrus activity and face recognition performance. Kim et al. (2010) found that participants given the dopamine precursor L-dihydroxypheylalanine (L-dopa) showed greater activation in the bilateral fusiform gyrus compared to participants assigned a placebo. Recently, Rypma et al. (2015) reported that local dopamine availability, as assessed using dopamine D1 binding potential, predicts neural activity in the fusiform gyrus during a face recognition task. A high blood-oxygen-level-dependent (BOLD) response relative to dopamine availability supported higher face recognition performance. Similarly, dopamine has been consistently implicated in the reward system (Taber et al., 2012) and faces can be rewarding stimuli (e.g., Stavropoulos and Carver, 2014). Reward associated with viewing faces may affect how memorable those faces are (Marzi and Viggiano, 2010).

The COMT enzyme metabolizes synaptic catecholamines, accounting for over 60% of the degradation of dopamine in the mammalian frontal cortex (Karoum et al., 1994). The gene coding for COMT contains a functional single nucleotide polymorphism (SNP) that affects the thermal stability and activity of the enzyme (Lotta et al., 1995; Lachman et al., 1996). The *COMT* val158met SNP involves a valine (val) being substituted for a methionine (met). Studies suggest the met allele decreases COMT activity by somewhere between 30% (Chen et al., 2004) and 67–75% (Lachman et al., 1996). Consequently, dopamine presumably remains active in the synapse for a longer duration, leading to enhanced dopamine signaling (Weinberger et al., 2001). The *COMT* alleles are co-dominant, and heterozygotes typically display an intermediate phenotype (Lachman et al., 1996; Chen et al., 2004). *COMT* heterozygotes (val/met) make up 46% of a European population, while 29% are val homozygotes (val/val) and 25% are met homozygotes (met/met; HapMap-CEU).

Maintenance of both *COMT* alleles in the population may be explained using the warrior/worrier dichotomy proposed by Goldman et al. (2005), in which the *COMT* val allele is associated with both stress resistance and poorer cognitive performance, while the evolutionarily more recent met allele confers cognitive advantages but also affective vulnerability. Research indicates the met allele is associated with better performance on tasks tapping working memory (Goldberg et al., 2003; Aguilera et al., 2008), processing speed (Bilder et al., 2002), and executive functions (Egan et al., 2001; Malhotra et al., 2002; Rosa et al., 2004). Despite these positive reports, studies on COMT genotype and cognition have produced inconsistent results. A meta-analysis of the effects of *COMT* genotype on a range of cognitive phenotypes reported no associations between *COMT* and any phenotypes other than IQ (Barnett et al., 2008). Barnett et al. (2008) suggest that early promising results and a publication bias may have contributed to potentially unwarranted enthusiasm concerning the effects of *COMT* on cognition. Between-study heterogeneity indicates that relationships between COMT and cognition may vary between populations, or as a consequence of a number of other population-independent variables (Barnett et al., 2008).

Sex-specific effects of the *COMT* polymorphism on cognition could contribute to inconsistencies in the literature, as these effects may be obscured when sex is not considered in the analysis. There is accumulating evidence of *COMT* genotype-sex interactions on a range of phenotypes, including psychiatric disorders (Harrison and Tunbridge, 2008), personality traits (Chen et al., 2011), and forms of cognition such as verbal ability and memory (O’Hara et al., 2006; Soeiro-De-Souza et al., 2013). Recently, *COMT* genotype was shown to interact with the α_2A_-receptor gene promotor (*ADRA2A* C-1291G) polymorphism and sex in affecting face perception (Tamm et al., 2016). There is some indication that the impact of *COMT* genotype on cognition may be stronger in males than in females (Barnett et al., 2007; Harrison and Tunbridge, 2008). An inverted “U”- shaped relationship is thought to exist between dopamine and cognitive performance, with cognition being impaired by suboptimal and supraoptimal dopamine levels (Mattay et al., 2003; Vijayraghavan et al., 2007). Factors such as genotype and sex may interact to influence an individual’s baseline position on this hypothetical curve (O’Hara et al., 2006).

Interactions between *COMT* genotype and sex may be at least in part due to regulatory effects of estrogen on dopaminergic transmission and COMT activity. Estrogen appears to facilitate the release and synthesis of dopamine (Becker, 1990, 2000; Xiao and Becker, 1994; Pasqualini et al., 1995) and may thus contribute to sex differences documented for cognitive phenotypes. Estrogen has been reported to decrease COMT mRNA levels and activity (Cohn and Axelrod, 1971; Xie et al., 1999; Jiang et al., 2003). Consistent with these down-regulation effects of estrogen on COMT, women with particularly high levels of estrogen (due to being in the third trimester of pregnancy or taking an oral contraceptive) show lower levels of COMT activity than other women (Briggs and Briggs, 1973). Furthermore, Chen et al. (2004) found that prefrontal COMT activity was around 17% higher for males than for females. This finding was independent of *COMT* val158met genotype. These higher levels of activity occur despite COMT protein and mRNA levels not differing between males and females (Bray et al., 2003; Chen et al., 2004; Tunbridge et al., 2004).

The present study builds upon research previously reported by Lamb et al. (2015), in which *COMT* val^158^met genotype did not affect face recognition performance in a smaller sample of young adults. The aim of the present study was to determine whether the *COMT* val^158^met polymorphism has sex-specific effects on face recognition performance that may be obscured in studies that do not consider genotype-sex interactions. Due to the effects of estrogen on COMT activity and face recognition performance, as well as previously documented *COMT* genotype-sex interactions, the *COMT* polymorphism was hypothesized to interact with sex to affect face recognition performance. We also predicted a significant main effect of sex on face recognition, replicating past studies.

## Material and Methods

### Participants

A sample of 142 university students aged between 18 and 42 years (*M* = 22.7, *SD* = 3.7; Supplementary Table A1) participated in the present study. Of these participants, 90 (63.4%) were female and 100 (70.4%) were participants from the study previously published by Lamb et al. (2015). All participants had either normal or corrected-to-normal vision. They had no learning disabilities or visual disorders and gave their informed consent for inclusion in this study. The University of Auckland Human Subjects Ethics Committee approved all study procedures.

### Genotyping

#### DNA Collection

Participants were requested to provide a small blood or saliva sample. Blood collection occurred under sterile conditions. Saliva samples were collected using Oragene-DNA Self Collection kits following the instructions of the manufacturer.

#### DNA Extraction

DNA extraction from the blood samples followed the method given by Miller et al. (1988), while extraction from saliva samples followed the method given by Nishita et al. (2009). DNA samples were then resuspended in Tris-EDTA buffer and quantified using Nanodrop ND-1000 1-position spectrophotometer (Thermo Scientific).

#### DNA Amplification

Samples of DNA were diluted to 50 ng/μL. DNA amplification was carried out following a modified version of that described by Erickson et al. (2008). Amplification of the 176 bp polymorphic *COMT* fragment used the primers COMT-F 5′-TCA CCA TCG AGA TCA ACC CC-3′ and COMT-R 5′-GAA CGT GGT GTG AAC ACC TG-3′. Polymerase chain reaction (PCR) used 10X Taq buffer (2.5 L μL), Taq polymerase (0.125 μL), dNTPs (5 nmol), primers (10 pmol each), Q solution (5 μL) and DNA (100 ng) made up to 25 μL with dH_2_O. Denaturation occurred at 95°C for 15 min. There was then 30 cycles on a ThermoCycler (involving denaturation at 94°C for 30 s, annealing at 60°C for 30 s, and extension at 72°C for 30 s), followed by a final extension at 72°C.

#### Enzyme Digestion

Polymerase chain reaction product (8 μL) was incubated with N1aIII for 1 h at 37°C. Analysis of digestion products used a high-resolution agarose gel (4%) with a Quick Load 100 bp ladder (BioLabs) and a GelPilot Loading Dye (QIAGEN). DNA was immersed in an ethidium bromide solution for 10 s and then was visualized under ultraviolet light.

#### Genotyping

Enzyme digestion resulted in bands of 82, 54 and 41 bp for the val^158^ allele and the 82 bp fragment was cut into 64 and 18 bp fragments for the met^158^ allele. Genotyping followed the method described by Erickson et al. (2008).

### Face Recognition Memory

Face recognition was assessed using the Faces I subtest of the Wechsler Memory Scale – Third Edition (WMS-III; Wechsler, 1997b). In the Faces task, participants were presented with a series of 24 faces, presented for 2 s each. They were requested to remember each face. Immediately after the presentation of this first series, they were serially presented with 48 images of faces. Twenty four of these were the previously encountered faces, while the 24 were new. Participants were asked to indicate whether they had previously encountered each face.

For young adults, the Faces I subtest have demonstrated split-half reliability coefficients in the range of 0.75–0.79 (Wechsler, 1997a), suggesting the measure has adequate internal consistency. The subtest has a test–retest stability coefficient of 0.70 for the 16–54 year age group, indicating an appropriate level of reliability (Wechsler, 1997a). While the Faces I subtest is not highly correlated with other measures of visual memory (e.g., Millis et al., 1999), this is likely to be due to the neural circuitry underlying familiarity-based recognition differing from that of recall (e.g., Aggleton and Brown, 1999).

### Data Analysis

#### Data Preparation

Raw scores out of 48 on the Faces subtest were converted into percentage of correct responses for analyses. Observed *COMT* genotypes were consistent with those predicted by Hardy Weinberg equilibrium (χ^2^ = 0.109, *p* > 0.05). Of the 142 participants, 38 (26.8%) were val homozygotes, 35 (24.6%) were met homozygotes and 69 (48.6%) were heterozygotes (val/met). Assumption testing on the data supported the use of parametric procedures.

#### Statistical Analyses

A two-way ANOVA was conducted on the face recognition scores, with *COMT* genotype (val/val; val/met; met/met) and sex as the between-subjects independent variables.

Mean face recognition scores by *COMT* genotype and sex are shown in Supplementary Table A2.

## Results

**FIGURE 1:**
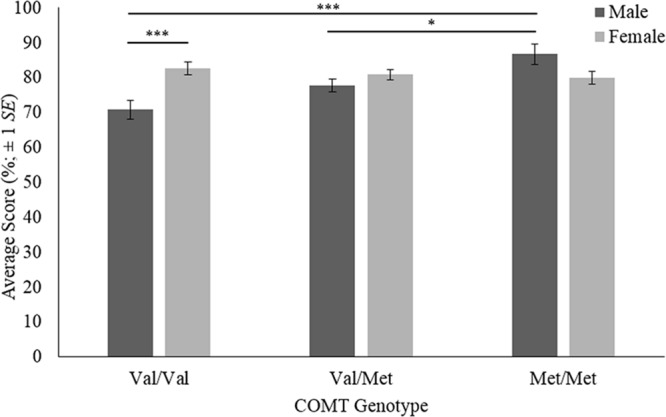
**Face recognition scores for COMT genotypes by sex.** The error bars are based on ±1 standard error. ^∗^*p* < 0.05, ^∗∗∗^*p* < 0.001.

Results of the two-way ANOVA are shown in Supplementary Table A3. There was a significant main effect of *COMT* genotype on face recognition [*F*_(2,136)_ = 3.862, *p* = 0.023, η^2^ = 0.049]. There was no significant main effect of sex (*p* > 0.05). There was, however, a significant two-way interaction between *COMT* genotype and sex on face recognition [*F*_(2,136)_ = 7.631, *p* = 0.001, η^2^ = 0.097; **Figure 1**).

Simple effects tests using Bonferroni adjustments were carried out on the significant two-way interaction. The effect of *COMT* genotype for each sex was analyzed first. Pairwise comparisons are displayed in Supplementary Table A4. Of the male participants, met homozygotes (*M* = 86.6, *SE* = 2.87) achieved significantly higher face recognition scores than both heterozygotes (*M* = 77.6, *SE* = 1.80; *p* = 0.028) and val homozygotes (*M* = 70.7, *SE* = 2.64; *p* < 0.001). The comparison between heterozygotes and val homozygotes was not significant (*p* < 0.05). For females, no differences between *COMT* genotypes were significant (*p* > 0.05). This suggests the main effect of *COMT* is best understood as an artifact of the interaction between genotype and sex. Secondly, the effect of sex for each *COMT* genotype was analyzed. Pairwise comparisons are shown in Table A5. For val homozygotes, females (*M* = 82.6, *SE* = 1.91) achieved significantly higher scores than males (*M* = 70.7, *SE* = 2.64; *p* < 0.001). For heterozygotes and met homozygotes, there were no significant sex differences in face recognition (*p* > 0.05).

## Discussion

Our results provide support for our hypothesis that *COMT* val^158^met genotype and sex would interact to affect memory for faces. The *COMT* polymorphism only affected face recognition performance in male participants. Male met homozygotes outperformed both the male heterozygotes and the male val homozygotes. While there was no significant difference between the male heterozygotes and the male val homozygotes, this may be due to low statistical power and a dosage effect trend does appear to be emerging. Our hypothesis that there would be a main effect of sex on face recognition performance was not supported, and a sex difference in face recognition ability was only found for *COMT* val homozygotes, with females attaining higher scores. While there was an unexpected main effect of *COMT* genotype on face recognition, this was not consistently observed across the sexes and is thus better understood as an artifact of the interaction between genotype and sex.

The presence of an interaction between *COMT* val^158^met genotype and sex on face recognition is consistent with other studies that have found sex to affect the impact of the *COMT* polymorphism on cognitive phenotypes (e.g., O’Hara et al., 2006; Barnett et al., 2007; Soeiro-De-Souza et al., 2013; Gurvich and Rossell, 2015). O’Hara et al. (2006) reported an interaction between *COMT* and sex on verbal memory, with male val homozygotes outperforming heterozygote and met homozygote men while females did not differ as a function of genotype. This effect of *COMT* genotype on memory in male participants is inconsistent with our study, where a met advantage was documented. This inconsistency may be due to the difference in the form of memory being assessed, as well as the effects of *COMT* on cognition varying over the lifespan. O’Hara et al. (2006) examined cognition in older adults. Age related declines in dopamine receptor densities may reduce the amount of dopamine that is optimal for cognitive performance (Harris et al., 2005), although studies of *COMT* and cognition in older adults do not consistently support this shift (e.g., Nagel et al., 2008; Josefsson et al., 2012).

Generally, studies with young adult samples that have found an association between *COMT* genotype and memory performance or executive function have reported higher scores in met allele carriers (Witte and Flöel, 2012). Our finding of a performance advantage in male met homozygotes relative to males with the val allele suggests that the lower levels of dopamine associated with the val allele are less conducive to face recognition. This fits with recent research linking dopamine to the functioning of the fusiform face area (Rypma et al., 2015). Furthermore, the effects of the COMT enzyme on dopamine are particularly pronounced in the prefrontal cortex (PFC; Chen et al., 2004) and neuroimaging research has implicated the lateral PFC of the right hemisphere in memory for faces (e.g., Druzgal and D’Esposito, 2003; Sergerie et al., 2005).

The male-specific effect of *COMT* genotype on face recognition is consistent with literature suggesting that some of the effects of *COMT* on cognition may be more pronounced in males (e.g., Barnett et al., 2007; Harrison and Tunbridge, 2008). In *COMT* knock-out mice, males show an increase in frontal dopamine while dopamine levels in females do not change (Gogos et al., 1998). Similarly, the effect of *COMT* genotype on COMT activity in human lymphocytes is stronger in males than in females (Chen et al., 2004). *COMT* genotype and enzyme activity may thus be less consequential for females, possibly due to hormone-regulated compensatory mechanisms in the neurotransmission or metabolism of catecholamines (Gogos et al., 1998).

Estrogen has been shown to inhibit the expression of *COMT* mRNA and reduce the activity of the COMT enzyme (Cohn and Axelrod, 1971; Xie et al., 1999; Jiang et al., 2003), as well as enhancing dopamine synthesis and release (Becker, 1990, 2000; Xiao and Becker, 1994; Pasqualini et al., 1995). This appears to contribute to sex differences in the dopaminergic system. Research on COMT activity and thermal stability in the human liver has demonstrated significantly lower levels of COMT activity in females compared to males (Boudikova et al., 1990). Similarly, COMT activity is lower for females than for males in the human PFC, independent of *COMT* genotype (Chen et al., 2004). In striatal and extrastriatal regions, levels of baseline dopamine and dopamine released in response to D-amphetamine are higher in women than men (Riccardi et al., 2006, 2011).

The current study demonstrated a sex difference in face recognition performance that was only present for *COMT* val homozygotes. Males have been shown to have higher levels of COMT activity than females in the human PFC, as well as for each individual genotype (Chen et al., 2004). Male val homozygotes thus have lower levels of synaptic dopamine than their female counterparts. This particular genotype-sex combination may be associated with dopamine levels that are sub-optimal for performance on the face recognition task. Interestingly, the forms of cognition for which O’Hara et al. (2006) found *COMT* genotype-sex interactions are also abilities for which sex differences tend to traditionally manifest (Herlitz and Rehnman, 2008). Social experiences may perpetuate or mitigate the effects of differing dopamine levels.

It is possible that social experiences during development could also render male val homozygotes more vulnerable than female val homozygotes to showing poorer face recognition. Compared to that of boys, the socialization of girls has tended to focus more on the promotion of emotional understanding and interpersonal sensitivity (McClure, 2000). This could encourage girls to pay greater attention to faces, which convey emotion, and boys may consequently have less cumulative experience with faces (McClure, 2000). Differences in socialization may amplify early hormone-related sex differences in behavior toward facial stimuli. Female infants of 12 months make significantly more eye contact with their parents than male infants of the same age (Lutchmaya et al., 2002). Research by Connellan et al. (2000) suggests that sex differences in preference for viewing faces over non-social objects may be present in neonates as early as 1 day after birth, with girls showing more interest in a face than a mobile (although see Alexander and Wilcox, 2012).

Male val allele carriers may also have less accumulated experience with faces than their met homozygote counterparts. Reduced availability of synaptic dopamine in *COMT* val homozygotes may disrupt the processing of reward information (Wichers et al., 2008; Dreher et al., 2009). This could result in the viewing of facial stimuli being less rewarding to val homozygotes and their attention to faces may thus be reduced. Over the course of development, impaired social reward could interact with environmental factors to contribute to male val homozygotes having poorer face recognition ability in young adulthood. Differences in dopamine levels and experience with faces may be protective in female val homozygotes. It is interesting to note that autism spectrum disorder, in which reduced sensitivity to social reward (e.g., Lin et al., 2012; Sepeta et al., 2012) has been linked to dysregulated dopaminergic activity (Mittleman and Blaha, 2015), occurs at higher rates in males than females (Baron-Cohen, 2002). While there has been some indication that the *COMT* val allele may increase susceptibility to autism (James et al., 2006), studies examining a potential relationship between *COMT* and autism thus far have been limited and yielded inconclusive and conflicting results (e.g., Guo et al., 2013).

Uncertainty concerning the mechanisms underlying the *COMT* genotype-sex interaction is a limitation of our research. These are likely to be multifactorial and may affect different phenotypes in differing ways (Harrison and Tunbridge, 2008). As our sample consisted of young adults, it would be interesting to see if the genotype-sex interaction on face recognition is present in older adults. Estrogen levels in postmenopausal women fall to below those of their male peers (Bjørnerem et al., 2004), and research on an older sample may thus help to elucidate the mechanisms underlying the interaction in our study.

In the current study, we were unable to control for factors such as menstrual cycle phase and use of oral contraceptives in female participants. Women taking oral contraceptives may have higher levels of COMT activity than other women (Briggs and Briggs, 1973). Further research on a larger sample could explore the potential effects of these variables on the interaction demonstrated in our study. Lastly, a limitation of our study was a relatively small sample size. This is particularly pertinent to consider given an interaction was being tested. Homozygotes were less frequently observed than heterozygotes and, when divided by sex, cell counts were low. There is a need for further large-*n* studies investigating the scope and nature of *COMT* genotype-sex interactions on cognition.

## Conclusion

The current study is believed to be the first to demonstrate a male-specific effect of the *COMT* val^158^met polymorphism on face recognition performance in young adults. Male met homozygotes demonstrated a greater ability to determine whether faces had been previously encountered than male val carriers. A more rapid deactivation of synaptic dopamine in male val allele carriers may drive this effect. This study thus contributes to accumulating evidence of sex-specific effects of COMT on cognitive phenotypes. Sex-specific effects of the *COMT* polymorphism are likely to be obscured when sex-genotype interactions are not considered in analyses. Studies failing to consider these interactions may have contributed to inconsistencies in research on *COMT* and cognition.

While previous research has demonstrated a female advantage for performance on face recognition tasks, our study found a sex difference in face recognition for *COMT* val homozygotes only. Female *COMT* val homozygotes outperformed males of this genotype. Due to the down-regulation of COMT activity by estrogen, males with the val/val genotype may have lower levels of dopamine than their female counterparts. This is consistent with a role for dopamine in our ability to recognize previously encountered faces. Hormones or social factors may interact with dopamine levels to facilitate performance in female val homozygotes. Future research may seek to explore potential sex differences in the effects of *COMT* genotype on PFC dopaminergic activity and how these concur with sex-specific effects on cognition.

## Author Contributions

YL was responsible for conception and study design. Data were collected by YL, NM, and SS. Data analysis was carried out YL, with KW as statistics advisor. The manuscript was written and prepared for submission by YL under the supervision of IJK.

## Conflict of Interest Statement

The authors declare that the research was conducted in the absence of any commercial or financial relationships that could be construed as a potential conflict of interest.
