# Supplementary material for: Catechol-O-methyltransferase val158met Polymorphism Interacts with Sex to Affect Face Recognition Ability
Source: Front Psychol. 2016 Jun 27;7:965. doi: 10.3389/fpsyg.2016.00965 (PMC4921451; doi:10.3389/fpsyg.2016.00965)
Supplement: Supplementary file 1 [file Data_Sheet_1.DOCX]

Table A1

*Mean Participant Age in Years by COMT Genotype and Sex.*

| Sex | COMT | Mean^a^ | Std. Dev. |
| --- | --- | --- | --- |
| Male | Val/Val | 22.2 | 2.4 |
|  | Val/Met | 23.1 | 3.6 |
|  | Met/Met | 23.9 | 6.4 |
|  | Total | 23.1 | 4.1 |
| Female | Val/Val | 22.8 | 3.3 |
|  | Val/Met | 21.9 | 3.2 |
|  | Met/Met | 23.2 | 4.3 |
|  | Total | 22.5 | 3.6 |
| Total | Val/Val | 22.5 | 3.0 |
|  | Val/Met | 22.5 | 3.4 |
|  | Met/Met | 23.5 | 4.9 |
|  | Total | 22.7 | 3.7 |

^a^ No group differences were significant (*p* > .0.5).

Table A2

*Mean Face Recognition Scores by COMT Genotype and Sex.*

| Sex | COMT | Mean | Std. Error | *N* |
| --- | --- | --- | --- | --- |
| Male | Val/Val | 70.7 | 2.64 | 13 |
|  | Val/Met | 77.6 | 1.80 | 28 |
|  | Met/Met | 86.6 | 2.87 | 11 |
|  | Total | 77.8 | 1.43 | 52 |
| Female | Val/Val | 82.6 | 1.91 | 25 |
|  | Val/Met | 80.8 | 1.50 | 41 |
|  | Met/Met | 79.86 | 1.95 | 24 |
|  | Total | 81.1 | 1.04 | 90 |
| Total | Val/Val | 78.5 | 1.63 | 38 |
|  | Val/Met | 79.5 | 1.17 | 69 |
|  | Met/Met | 82.0 | 1.74 | 35 |
|  | Total | 79.9 | .85 | 142 |

Table A3

*Two-way ANOVA for Face Recognition Scores with COMT Genotype and Sex as the Between-Subjects Variables*

| Source | *F* | df | *p* |
| --- | --- | --- | --- |
| Sex | 2.536 | 1 | .114 |
| COMT Genotype | 3.862* | 2 | .023 |
| COMT*Sex | 7.631** | 2 | .001 |
| Error |  | 136 |  |

**p* < .05, ** *p* < .01

Table A4

*Pairwise comparisons for COMT genotype at each level of sex.*

| Sex | Comparison | Mean difference | Std. Error | *p* |
| --- | --- | --- | --- | --- |
| Male | Val/Val – Val/Met | -6.9 | 3.20 | .096 |
|  | Val/Val – Met/Met | -15.9 | 3.91 | .000 |
|  | Val/Met – Met/Met | -8.9 | 3.39 | .028 |
| Female | Val/Val – Val/Met | 1.8 | 2.42 | 1.000 |
|  | Val/Val – Met/Met | 2.7 | 2.72 | .958 |
|  | Val/Met – Met/Met | 1.0 | 2.45 | 1.000 |

Table A5

*Pairwise comparisons for sex at each level of COMT genotype.*

| COMT | Comparison | Mean difference | Std. Error | *p* |
| --- | --- | --- | --- | --- |
| Val/Val | Male – Female | -11.9 | 3.26 | .000 |
| Val/Met | Male – Female | -3.2 | 2.34 | .170 |
| Met/Met | Male – Female | 6.7 | 3.47 | .056 |
